# Exploring the Zoonotic Potential of *Mycobacterium avium* Subspecies *paratuberculosis* through Comparative Genomics

**DOI:** 10.1371/journal.pone.0022171

**Published:** 2011-07-22

**Authors:** James W. Wynne, Tim J. Bull, Torsten Seemann, Dieter M. Bulach, Josef Wagner, Carl D. Kirkwood, Wojtek P. Michalski

**Affiliations:** 1 Australian Animal Health Laboratory, CSIRO Livestock Industries, Geelong, Victoria, Australia; 2 Department of Cellular and Molecular Medicine, St. George's Hospital Medical School, Cranmer Terrace, London, United Kindgom; 3 Victorian Bioinformatics Consortium, Monash University, Clayton, Victoria, Australia; 4 Department of Paediatrics, University of Melbourne, Enteric Virus Group, Murdoch Childrens Research Institute, Royal Children's Hospital, Parkville, Victoria, Australia; Institute of Microbial Technology, India

## Abstract

A comparative genomics approach was utilised to compare the genomes of *Mycobacterium avium* subspecies *paratuberculosis* (MAP) isolated from early onset paediatric Crohn's disease (CD) patients as well as Johne's diseased animals. Draft genome sequences were produced for MAP isolates derived from four CD patients, one ulcerative colitis (UC) patient, and two non-inflammatory bowel disease (IBD) control individuals using Illumina sequencing, complemented by comparative genome hybridisation (CGH). MAP isolates derived from two bovine and one ovine host were also subjected to whole genome sequencing and CGH. All seven human derived MAP isolates were highly genetically similar and clustered together with one bovine type isolate following phylogenetic analysis. Three other sequenced isolates (including the reference bovine derived isolate K10) were genetically distinct. The human isolates contained two large tandem duplications, the organisations of which were confirmed by PCR. Designated vGI-17 and vGI-18 these duplications spanned 63 and 109 open reading frames, respectively. PCR screening of over 30 additional MAP isolates (3 human derived, 27 animal derived and one environmental isolate) confirmed that vGI-17 and vGI-18 are common across many isolates. Quantitative real-time PCR of vGI-17 demonstrated that the proportion of cells containing the vGI-17 duplication varied between 0.01 to 15% amongst isolates with human isolates containing a higher proportion of vGI-17 compared to most animal isolates. These findings suggest these duplications are transient genomic rearrangements. We hypothesise that the over-representation of vGI-17 in human derived MAP strains may enhance their ability to infect or persist within a human host by increasing genome redundancy and conferring crude regulation of protein expression across biologically important regions.

## Introduction

*Mycobacterium avium* subspecies *paratuberculosis* (MAP), a Gram-positive acid fast bacillus, is a member of the *Mycobacterium avium* complex and is the causative agent of Johne's disease (JD), a chronic granulomatous enteritis affecting ruminants. While there is no doubt MAP has the ability to cause enteric disease in animals its potential zoonotic role in human conditions, such as Crohn's disease (CD), remains unresolved. The first isolation of viable MAP from a CD patient was made almost 25 years ago [1], [2]. Kirkwood et al. [3] more recently demonstrated that MAP could be identified by IS*900* PCR significantly more often in mucosal biopsies and or peripheral blood mononuclear cells (PBMCs) from paediatric CD patients (47%) not yet receiving therapy, when compared to non-IBD controls (11%). Yet viable MAP could only be cultured from mucosal biopsies from four of ten CD patients and none of the non-IBD controls.

MAP is an extremely persistent pathogen that can survive within the livestock environment (i.e., water, faeces and soil) for long periods [4], [5]. While bacilli from these environmental sources may pose some risk to humans, the main source of transmission from animals to humans is more likely to be via contaminated milk. A study of 567 pasteurised milk samples from the UK found 11.8% were MAP positive by PCR analysis and that MAP could be cultured from 1.8% [6]. Similar recovery rates have been found elsewhere [7] which indicate a possible transmission route of live MAP from animals to humans is occurring through contaminated milk and possibly through animal derived foodstuff.

Due to the importance of MAP as a global animal pathogen and its potential zoonotic role in CD, many studies have investigated the genetic diversity of MAP isolated from different host species. A number of strategies have been developed for assessing the genetic variation of MAP isolates. Restriction fragment length polymorphism (RFLP) [8] was the strategy first utilised and it demonstrated the existence of three animal derived strain types. Other techniques such as PCR-restriction endonuclease analysis of the insertion sequence IS*1311* [9], IS*900* RFLP [10], pulsed-field gel electrophoresis [11], representational difference analysis [12] candidate gene analysis [13], [14], [15] and, most recently, comparative genome hybridisation [16], [17], [18], [19], [20], [21] have confirmed the existence of these three MAP types. Each strain type contains varying degrees of genomic deletions derived from a putative MAP precursor genome. Type I MAP strains predominantly infect ovine hosts, whilst Type II principally infect bovine hosts. Type III MAP has been isolated from both ovine, bovine and caprine sources [4]. Previous genetic investigations have shown MAP strains isolated from humans cluster with strains of bovine origin [19], [22]. AFLP fingerprinting however has suggested bovine MAP cluster into two major nodes but those recovered from sheep or humans resolve on separate branches [23].

To date, the complete genome sequence is available for only one MAP isolate [24], [25]. This isolate, a bovine derived Type II strain (designated K10), has served as an important reference genome for many genomic MAP studies. However, to gain an understanding of the broader genomic diversity within this species, multiple strains must be sequenced. This is particularly relevant for MAP due to the significant genetic differences observed between the three major strain types. The comparison of multiple strains from a single species is now common practice [26], [27]. Indeed, multiple *Mycobacterium tuberculosis* genomes have been fully sequenced and this has led to the identification of numerous genetic polymorphisms that may underline the basis of virulence attenuation in this species [28].

This study utilised high throughput DNA sequencing combined with comparative genome hybridisation to examine the genetic relationship between multiple human and animal derived MAP strains at a genome-wide level. Genetic differences between strains may reveal phylogenetic relationships that provide a better understanding of the processes involved with MAP zoonotic transmission.

## Materials and Methods

### MAP isolates

The genomes of ten MAP isolates (seven human and three animal derived) were sequenced using the Illumina GAIIx platform. The details of each isolate are presented in Table 1. The seven human isolates were cultured from mucosal biopsies taken from the ileum or caecum from paediatric patients undergoing initial endoscopy at the Royal Children's Hospital (Melbourne, Australia) between 2003 and 2008. Diagnosis of IBD was established using standard clinical, endoscopic and histopathological criteria as reported previously [29]. For the purpose of this study clinicians diagnosed patients as CD, UC or non-IBD and researchers were informed of the diagnosis prior to MAP isolation. The initial isolation of human CD derived isolates Pt139, Pt144, Pt145 and Pt149 was conducted and published by Kirkwood et al. [3]. Subsequent isolation of strains from non-IBD (Pt154, Pt155) and UC (Pt164) patients were conducted by us exactly as described by Kirkwood et al. [3]. The collection of all mucosal biopsies was approved by the Human Ethics Committee of the Royal Children's Hospital (EHRC no. 23003). Informed consent was obtained from each individual parent or guardian [3].

**Table 1:** Details of *Mycobacterium avium* subspecies *paratuberculosis* isolates investigated in this study.

Isolate	Type	Host	Sample	Origin	Diagnosed condition	Ref
CLIJ623‡	II	bovine	gut autopsy	Australia	clinical BJD	[31]
CLIJ644‡	II	bovine	gut autopsy	Australia	clinical BJD	-
CLIJ361‡	I	ovine	gut autopsy	Australia	clinical OJD	[31]
Pt139‡	II	human	gut biopsy	Australia	early onset CD	[3]
Pt144‡	II	human	gut biopsy	Australia	early onset CD	[3]
Pt145‡	II	human	gut biopsy	Australia	early onset CD	[3]
Pt146‡	II	human	gut biopsy	Australia	early onset CD	[3]
Pt154‡	II	human	gut biopsy	Australia	Non-IBD gastritis	-
Pt155‡	II	human	gut biopsy	Australia	Non-IBD	
spirochaetosis	-					
Pt164‡	II	human	gut biopsy	Australia	UC	-
ATCC-19698	II	bovine	faeces	USA	clinical BJD	[50]
ATCC-43544	II (Ben)	human	gut biopsy	USA	CD	[1]
ATCC-43015	II (Linda)	human	gut biopsy	USA	CD	[1]
CLIJ684	II	bovine	gut autopsy	Australia	clinical BJD	-
CLIJ748	II	bovine	gut autopsy	Australia	clinical BJD	-
96/1400-1	II	bovine	faeces	Australia	clinical BJD	-
93/6428	II	bovine	faeces	Australia	clinical BJD	-
96/4651	II	bovine	gut tissue	Australia	clinical BJD	-
96/5141	II	bovine	gut tissue	Australia	clinical BJD	-
97/5541-1	I	ovine	gut tissue	Australia	clinical OJD	-
98/3368	I	ovine	faeces	Australia	clinical OJD	-
99/3759-2	I	ovine	faeces	Australia	clinical OJD	-
99/340	I	ovine	gut autopsy	Australia	clinical OJD	-
K11	II	bovine	faeces	Wales, UK	clinical BJD	-
K18	II	bovine	faeces	Wales, UK	clinical BJD	[30]
K43	II	bovine	faeces	Wales, UK	clinical BJD	-
K57	II	bovine	faeces	Wales, UK	clinical BJD	-
K46	II	bovine	faeces	Wales, UK	clinical BJD	-
K47	II	bovine	faeces	Wales, UK	clinical BJD	-
K48	II	bovine	faeces	Wales, UK	clinical BJD	-
SN8	II	human	gut biopsy	USA	CD	[30]
US23	II	bovine	faeces	USA	clinical BJD	-
456	II	caprine	gut biopsy	Spain	clinical JD	[16]
464	II	caprine	gut biopsy	Spain	clinical JD	[16]
213G	I pigmented	ovine	gut biopsy	Scotland, UK	clinical JD	[10]
W43	II	-	river water	Wales, UK	-	[51]
S5	Bison	caprine	faeces	India	clinical JD	[52]
G50	II	bovine	faeces	Germany	clinical BJD	-
G128	II	bovine	faeces	Germany	clinical BJD	-
DJ1	II	bovine	faeces	Wales, UK	clinical BJD	-
CAM87	III	caprine	faeces	Spain	clinical JD	[16]

‡ represent isolates used for genome sequencing. CD = Crohn's disease, UC = ulcerative colitis, BJD = bovine Johne's disease, OJD = ovine Johne's disease.

Human CD MAP isolates ATCC-43544 (Ben) and ATCC-43015 (Linda) were obtained from the American Type Culture Collection (ATCC). Isolation and characterisation of these isolates has been described elsewhere [1], [2]. The human CD MAP isolate SN8 was a gift from Dr Saleh Naser of the University of Central Florida, USA. This isolate was obtained from an ileum biopsy from a CD patient in 2002 and was further characterised by Overduin et al. [30].

Strains labelled CLIJ were isolated from the ileocaecal valve from autopsied animals diagnosed with clinical JD from Victoria, Australia between 1997 and 2005. Collection and characterisation of CLIJ623 and CLIJ361 was performed by Stewart et al. [31]. Isolation and characterisation of isolates CLIJ644, CLIJ684 and CLIJ748 was performed exactly as described by Stewart et al. [31] and has not been published to date. Collection of these isolates was approved by the Australian Animal Health Laboratory Animal Ethics Committee (AAHL AEC no. 626). Isolation and characterisation of strains derived from caprine hosts (CAM87, 464 and 456) have been previously reported by Castellanos et al. [16].

The bovine strains: 96/1400-1, 93/6428, 96/4651 and 96/5141, and ovine strains: 97/5541-1, 98/3368, 99/3759-2 and 99/340, were isolated from faecal or tissue samples submitted to the Department of Primary Industries (Victoria) for routine of JD surveillance and diagnosis between 1993 and 1999. The bovine derived strains labelled K, and the single isolate DJ1, were isolated by the Veterinary Laboratory Agency (South Wales, UK) from faecal samples collected from animals with clinical JD or from animals in which subclinical MAP infection was suspected. These faecal samples were collected between 2000 and 2005. Strains labelled G were also isolated from faecal samples derived from animals with clinical JD (between 1999 and 2001) and were a gift from Dr Detlef Jonas of the Regional Veterinary Laboratory (Landesuntersuchungsamt Rheinland-Pfalz), Germany. The US23 strain was derived from a bovine host with clinical JD in the USA. To our knowledge a description of these strains has not been reported previously.

All isolates used for genome sequencing (including the ovine derived isolate CLIJ361) were cultured on Herrold's slants containing mycobactin, sodium pyruvate and fungizone for between six to nine months. All isolates were derived from a single colony. Ziehl-Neelsen staining was performed on each cultured isolate to confirm acid-fast bacilli.

### Genomic DNA isolations for genome sequencing, PCR and real-time PCR

DNA for whole genome sequencing was extracted using the chloroform extraction protocol as described herein. Bacterial cells were scraped from Herrold's slopes using a sterile loop and resuspended in 500 µl of TE (pH 8.0). Cells were washed once and resuspended in 500 µl of TE (pH 8.0). The cell suspension was first incubated at 80°C for 20 min and then at 37°C for 1.5 h containing 1 mg/ml of lysozyme (Sigma). RNaseA (Sigma) was added to a final concentration of 50 µg/ml and the cell lysate was incubated for a further 30 min at 37°C. The lysate was then incubated at 65°C for 20 min containing 1% SDS and 180 µg/ml of proteinase K (Sigma). CTAB (hexadecyltrimethyl ammonium bromide) to a final concentration of 1% (w/v) and NaCl to 0.7 M were added and the lysate incubated at 65°C for a further 10 min. Seven hundred and eighty microlitres of chloroform/isoamyl alcohol (24∶1) was then added, mixed and the sample centrifuged at 17,000×*g* for 5 min. Between 600–700 µl of the aqueous phase was removed and precipitated with 0.6 volumes of isopropanol at −20°C. The DNA was pelleted by centrifugation at 17,000×*g* for 15 min at 4°C. The pellet was washed once with 500 µl of 70% ethanol at 17,000×*g* for 10 min. The pellet was air dried for 10 min at room temperature and resuspended in nuclease free water.

All additional PCR assays, except the IS*900* PCR and real-time PCR, were performed using a crude DNA template as previously described by Moravkova et al. [32]. Briefly, a loop full of bacilli were scraped from a single Herrold's slope and resuspended in 50 µl of nuclease free water. The cell suspension was heated to 100°C for 20 min and centrifuged for 5 min at 17,000×*g*. The supernatant was transferred to a fresh tube and stored at −20°C until use.

### IS900 PCR

Amplification of the IS*900* element was performed for each isolate to confirm MAP identity prior to whole genome sequencing. PCR was conducted in a total volume of 50 µl containing 5 µl of 10× buffer (Qiagen), 200 µM of each dNTP (Promega), 0.2 µM of forward primer (JF11) and 0.2 µM of reverse primer (JF12) (Table S1), 2.5 units of *Taq* DNA polymerase and 5 µl of a 1∶100 diluted DNA template. Reactions were first subjected to 94°C for 3 min followed by 35 cycles of 94°C for 1 min, 58°C for 1 min and 72°C for 30 s. Amplicons were visualised on 2% TAE gels containing 1× GelRed stain (Biotium). PCR and restriction enzyme analysis of the IS*1311* element was previously used to confirmation animal isolates as Type I or Type II strains [31].

### Genome sequencing and reference assembly

For isolates CLIJ623, CLIJ644, CLIJ361, Pt139, Pt146, Pt154 and Pt164 whole genome sequencing was performed on the Illumina GAIIx platform using one flow-cell lane per isolate with 36-cycle paired-end chemistry. For isolates Pt144, Pt145 and Pt155 whole genome sequencing was performed with 70-cycle single-read chemistry. Reads were trimmed from the 3′ end to ensure a minimum Phred quality of 3, and read pairs containing ambiguous bases were removed. Read mapping onto the K10 genome sequence was performed using SHRiMP (ver. 1.3.2) [33] and SNPs and indels were called using Nesoni (ver. 0.29) (http://www.bioinformatics.net.au/software.shtml) with default parameters. Only SNPs for which the base was identified in greater than 80% of the mapped reads, with sequence coverage over 10-fold, were considered to represent unambiguous core SNPs. A recently revised version of the K10 genome was used as the reference genome [25].

### Comparative genomic hybridisation (CGH)

Microarray design and validation has been previously described for the MAPAC array [16]. MAP DNA for CGH analysis was extracted as described previously [16]. Briefly, approximately 10^9^ cells were scraped and emulsified through a 25-gauge needle in 650 µl mycobacterial lysis buffer (8.6 ml water, 0.5 M EDTA, 5 M NaCl, 1 M Tris-HCl [pH 8.0], 10% sodium dodecyl sulphate [SDS], 1 mg/ml lysozyme [Sigma]), 0.15 mg/ml proteinase K (Sigma), and 0.5 mg/ml lipase (Sigma, UK) then incubated at 37°C in a rotator for 1 h. Samples were added to lysing matrix B (MP Biomedicals, UK) in 1.9-ml ribolyser reaction tubes, mechanically disrupted in a FastPrep-24 ribolyser (MP Biomedicals) at 6,500 rpm for 45 s then kept on ice for 10 min. Lysate (220 µl) was added to 200 µl of Qiagen DNAeasy AL lysis buffer, mixed and applied to a DNAeasy column. Ethanol (100%; 200 µl) was then added and the tube sealed and mixed. Columns were washed in 500 µl Qiagen lysis buffers 1 and 2, with centrifugation at 8,000×*g* for 1 min, and then eluted in 90 µl DNA/RNase-free water overnight on the column at 4°C. MAP DNA from each sample (1.5 µg) was then labelled by random priming with 5 U Klenow polymerase (Invitrogen) to incorporate either Cy3 or Cy5 dCTP (GE Healthcare) for the test strain or the reference strain, respectively. Equal amounts of the Cy3- and Cy5-labelled samples were then co-purified through a Qiagen MinElute column, mixed with 500 µl Qiagen Buffer PB, washed twice with 500 µl Qiagen Buffer PE then eluted in 17 µl DNAse/RNAse free dH_2_O. Labelled DNA was then denatured at 95°C for 2 min in hybridisation buffer (30% formamide, 3.75× Denhardt's solution, 6× SSC, 0.75 mM sodium pyrophosphate, 37 mM Tris [pH 7.4], 0.075% SDS), loaded onto prehybridized (3.5× SSC [1× SSC is 0.15 M NaCl plus 0.015 M sodium citrate], 0.1% SDS, 10 mg/ml bovine serum albumin) microarray under two 22- by-22-mm LifterSlips (Erie Scientific), sealed in a humidified hybridization cassette (Corning) and hybridized overnight by immersion in a water bath at 55°C for 16 to 20 h. Slides were washed once in 400 ml 1× SSC, 0.06% SDS at 55°C for 2 min and twice in 400 ml 0.06× SSC for 2 min. Microarrays were then scanned using an Affymetrix 428 scanner, and signal intensity data were extracted using BlueFuse for Microarrays v3.5 (BlueGnome). Intensity data were post-processed by BlueFuse to exclude both controls and low-confidence data (*p*<0.1) prior to normalization by two-dimensional Lowess (window size of 20) and median centring. Further analysis of the normalized data was undertaken using GeneSpring 7.3.1 (Agilent Technologies). Analysis methods used triplicate microarray data from each strain with a hidden Markov model for CGH calling [34]. Genes showing >1.5 fold increase or decrease in signal over the control MAP K10 were listed as significant.

### Validation and characterisation of vGI duplications

PCR and sequencing was used to confirm genomic organisation of two apparent duplications (vGI-17 and vGI-18). We designed outward-facing primers (Table S1) located at the 5′ and 3′ ends of the duplicated region and performed PCR using genomic DNA as template. The vGI-17 PCR was performed in a total volume of 50 µl containing 25 µl of GoTaq Hot Start Green Master Mix (Promega), 0.2 µM of forward primer (GSP1-5′-vGI-17) and 0.2 µM of reverse primer (GSP1-3′-vGI-17), 5 µl of crude DNA template and nuclease free water to 50 µl. Reactions were first heated to 95°C for 3 min followed by 30 cycles of 95°C for 1 min, 58°C for 1 min and 72°C for 4 min. PCR for vGI-18 was performed in a total volume of 50 µl containing 1× *PfuUltra* II reaction buffer (Stratagene), 0.2 µM of forward primer (GSP1-5′-vGI-18) and 0.2 µM of reverse primer (GSP1-3′-vGI-18), 250 µM of each dNTP, 1 µl of *PfuUltra* II fusion HS DNA polymerase and 5 µl of crude DNA template. Reactions were subjected to 95°C for 2 min followed by 30 cycles of 95°C for 20 s, 62°C for 20 s and 72°C for 3 min, followed by a final extension for 3 min at 72°C. PCR products were resolved on 1% TAE agarose gel containing 1× GelRed DNA stain and visualised under UV transillumination. A selection of PCR products was purified using the QIA Quick PCR Clean-up Kit (Qiagen) and directly sequenced on both strands using the Applied Biosystems PRISM BigDye Terminator Mix and the Applied Biosystems 3730S Genetic Analyser (Applied Biosystems).

The vGI-17 duplication was quantified by real-time PCR using the standard curve method. MAPK_3057 was chosen as the endogenous control because this locus showed no evidence of duplication within any genome. We therefore assumed that a single copy of MAPK_3057 represents a single bacillus. CGH data also suggests that vGI-17 is present in no more than duplicate copies in any one cell. Absolute copy numbers of the vGI-17 and MAPK_3057 were then determined and ratio of vGI-17 to MAPK_3057 calculated. This ratio is interpreted as a percentage of cells that contain the vGI-17 duplication.

A total of 11 isolates were chosen for real-time PCR analysis and included the human isolates Pt139, Pt146, Pt154, Pt164, ATCC-43544 and the bovine isolates CLIJ623, CLIJ644, 96/1400, 93/6428, 96/4651 and 96/5141. For isolates Pt139, Pt146, Pt154, Pt164, ATCC-43544, CLIJ623 and CLIJ644 bacteria were harvested from Herrold's slants after approximately 20 months growth. For isolates 96/1400, 93/6428, 96/4651 and 96/5141 bacteria were harvested from Middlebrook 7H9 broth after 3 months of culture. DNA was extracted using the CTAB method as described above. Genomic DNA was quantified using the Qubit Fluorometer (Invitrogen) as per the manufacturer's recommendations.

First PCR products of vGI-17 and the endogenous control gene MAPK_3057 were produced as described above. MAPK_3057 PCR conditions were identical to vGI-17. PCR products were cloned into P-GEM-T Easy vector system (Promega), transformed into chemically competent *E.coli* (Bioline) and cultured on LB agar overnight at 37°C. Colonies were picked and further cultured overnight in LB broth. Plasmids were purified using the QIAprep Spin Miniprep kit (Qiagen). A serial dilution of plasmid DNA ranging from 10^1^ to 10^8^ copies was used to generate a standard curve. PCR conditions were identical for both vGI-17 and MAPK_3057 and were performed in triplicate. PCR was performed in a total volume of 20 µl containing 10 µl of EXPRESS SYBR GreenER™ qPCR SuperMix (Invitrogen), 0.2 µM of forward primer (GSP1-5′-vGI-17 or MAPK_3057F) and 0.2 µM of reverse primer (GSP1-3′-vGI-17 or MAPK_3057R), 0.4 µl of ROX Reference Dye (Invitrogen), 5 µl of plasmid template (or 50 ng of genomic DNA extracted from each isolate) and nuclease free water to 20 µl. Reactions were first heated to 95°C for 2 min followed by 40 cycles of 95°C for 30 s, 55°C for 30 s and 72°C for 3 min. The default melt curve analysis was conducted after the final cycle. PCR and data analysis was performed on an Applied Biosystems StepOnePlus real-time PCR system.

## Results

### Single nucleotide polymorphisms

**Figure 1:**
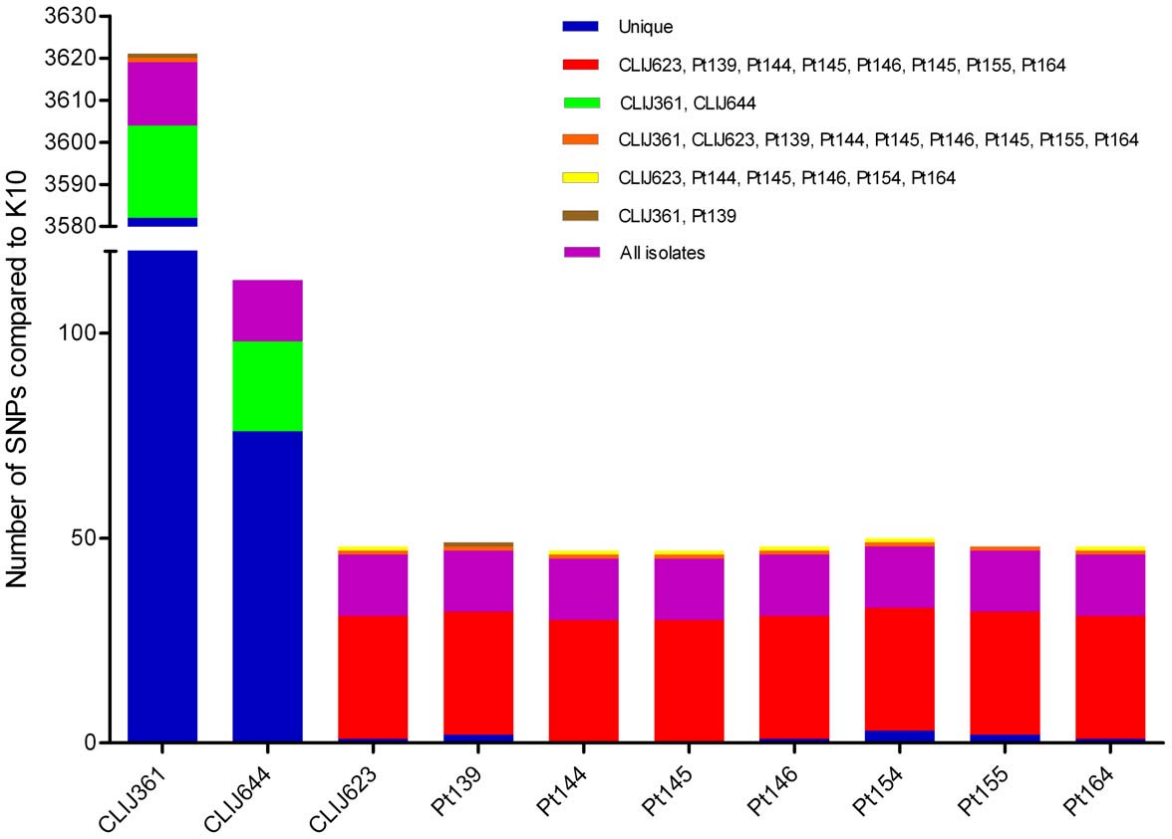
The number of SNPs identified in each isolate compared to the K10 reference genome. Identical colours represent SNPs shared between isolates. CLIJ = clinical John's disease, Pt = human patient. Note scale change on the y-axis. Origins of isolates are presented in Table 1.

Read mapping onto the corrected K10 genome [25] was used to detect single nucleotide polymorphisms (SNPs) within each isolate. Read mapping identified a total of 3738 sites across the K10 genome that sequence varied in one or more of the isolates sequenced in this study. The number of SNPs detected varied between isolates (Figure 1). The majority of these SNPs (3582; 95.8%) were unique to the CLIJ361 ovine isolate. A set of 30 SNPs were found to be shared by all human derived isolates and the bovine derived isolate CLIJ623. A set of 15 SNPs were found to be shared by all isolates compared to the K10 reference, possibly reflecting an Australian MAP specific polymorphism. A full description of SNPs identified within each isolate is presented in Table S2. All raw sequence data has been deposited in the NCBI Sequence Read Archive as study SRA030663. The Whole Genome Shotgun project has been deposited at DDBJ/EMBL/GenBank under the accession AFHX00000000 (CLIJ623), AFNR00000000 (CLIJ644), AFNS00000000 (CLIJ361), AFPC00000000 (Pt139), AFPD00000000 (Pt144), AFPE00000000 (Pt145), AFPF00000000 (Pt146), AFPG00000000 (Pt154), AFPH00000000 (Pt155) and AFPI00000000 (Pt164). The version described in this paper is the first version AFHX01000000, AFNR01000000, AFNS01000000, AFPC01000000, AFPD01000000, AFPE01000000, AFPF01000000, AFPG01000000, AFPH01000000 and AFPI01000000.

Although CLIJ644 is a typical bovine derived isolate it shared 22 SNPs with the ovine derived isolate CLIJ361 and 76 unique SNPs. Of particular note is the fact that CLIJ644 and CLIJ623, both Victorian bovine derived MAP isolates, demonstrated significant variation in SNP profiles (Figure 1). These isolates shared no common SNPs apart from the 15 SNPs identified in all sequenced Australian isolates.

**Figure 2:**
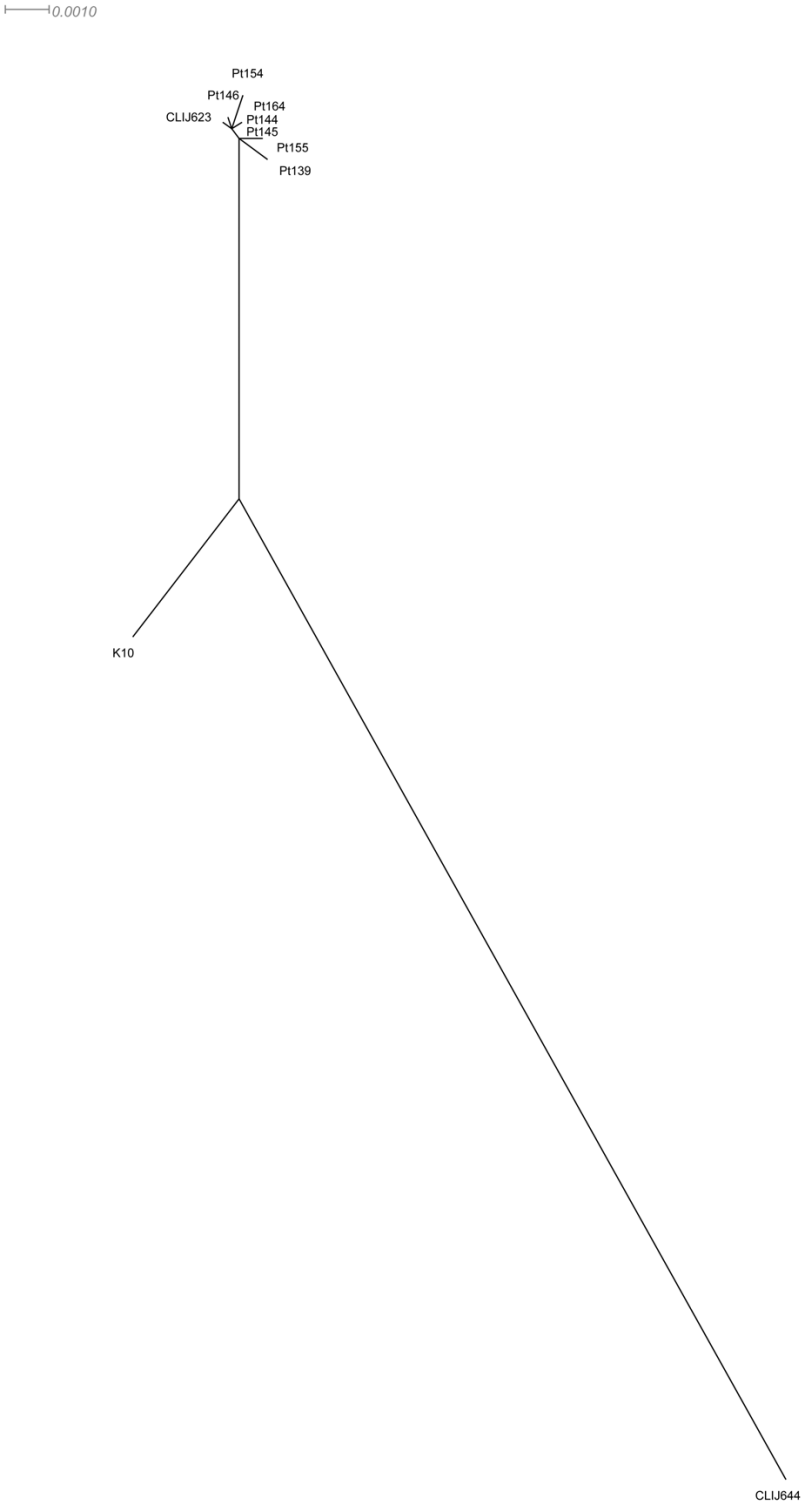
Phylogenetic analysis of human and bovine derived *Mycobacterium avium* subspecies *paratuberculosis*. Neighbour-joining phylogenetic tree based on single nucleotide polymorphisms (SNPs) of *Mycobacterium avium* subspecies *paratuberculosis* isolates derived from animal and bovine hosts. The ovine derived isolate CLIJ361 was omitted from this analysis due to its significant genetic divergence from the other isolates.

Phylogenetic analysis of bovine and human derived isolates based on SNPs revealed that human derived isolates show significant differences to both bovine CLIJ644 and K10 reference genomes, but are similar to CLIJ623 (Figure 2). The ovine derived isolate CLIJ361 was omitted from phylogenetic analysis due to its significant genetic divergence from the other isolates.

A total of 24 non-synonymous SNPs, including three deletions, three insertions and 18 substitutions, were identified within unique human derived isolates and CLIJ623 compared with K10. Seven open reading frames (ORFs) were either extended or truncated as a result (Table 2). It is perhaps most interesting to note that MAP isolates derived from CD and UC patients as well as the two non-IBD control subjects were highly similar.

**Table 2:** Functional consequence of non-synonymous SNPs.

Position	SNP	Nucleotide Change	Locus tag	Old length (aa)	New length (aa)	Product	Amino acid change	CLIJ623	Pt139	Pt144	Pt145	Pt146	Pt154	Pt155	Pt164
155742	deletion	T	MAPK_0117	406	257	acyl-CoA dehydrogenase FadE3_2	Truncates by 149 aa	+	+	+	+	+	+	+	+
388900	substitution	A→G	MAPK_0325	585	585	succinate dehydrogenase (flavoprotein subunit) SdhA	F→S	+		+	+	+	+	+	+
648289	substitution	G→A	MAPK_0556	629	629	NADH dehydrogenase I (chain L) NuoL	A→V	+	+	+	+	+	+	+	+
757950	substitution	C→G	MAPK_0662	348	348	DNA polymerase IV	Q→H	+	+	+	+	+	+	+	+
815870	substitution	G→C	MAPK_0716	201	201	transcriptional regulator, TetR family	G→R	+	+	+	+	+	+	+	+
858884	substitution	A→G	MAPK_0755	297	297	dehydrogenases	K→R		+						
968225	deletion	C	MAPK_0857	180	381	conserved hypothetical protein	Extends by 201 aa	+	+	+	+	+	+	+	+
1244576	substitution	C→G	MAPK_1136	122	122	GntR family transcriptional regulator	I→M						+		
2069337	substitution	T→G	MAPK_1828	361	361	transmembrane cytochrome C oxidase (subunit II) CtaC	L→R	+	+	+	+	+	+	+	+
2186283	substitution	G→C	MAPK_1923	185	185	conserved hypothetical membrane protein	K→N							+	
2467992	substitution	G→A	MAPK_2184	774	774	ATP-dependent protease La	G→D	+	+	+	+	+	+	+	+
2919070	substitution	T→G	MAPK_2538	407	407	uncharacterized protein conserved in bacteria	I→L	+	+	+	+	+	+	+	+
2983607	substitution	G→A	MAPK_2599	936	936	phosphoenolpyruvate carboxylase	P→S	+	+	+	+	+	+	+	+
3191834	substitution	C→T	MAPK_2788	290	290	dehydrogenases	A→V	+	+	+	+	+	+	+	+
3529711	substitution	A→C	MAPK_3110	341	341	acyl-[acyl-carrier protein] desaturase DesA1	M→L		+						
4041109	substitution	G→C	MAPK_3602	535	535	conserved membrane protein	A→G						+		
4150482	substitution	G→A	MAPK_3714	263	263	oxidoreductase	D→N	+	+	+	+	+	+	+	+
4279936	substitution	A→G	MAPK_3827	442	442	UDP-glucose 6-dehydrogenase	N→S	+	+	+	+	+	+	+	+
4385536	insertion-before	GC	MAPK_3924	389	135	carboxylate-amine ligase	Truncates by 254 aa	+	+	+	+	+	+	+	+
4406658	insertion-before	C	MAPK_3946	600	659	integral membrane C-type cytochrome biogenesis protein DipZ	Extends by 59 aa	+	+	+	+	+	+	+	+
4565931	insertion-before	G	MAPK_4093	575	195	exodeoxyribonuclease V (alpha chain), RecD	Truncates by 380 aa	+	+	+	+	+	+	+	+
4575454	substitution	G→A	MAPK_4099	258	227	conserved hypothetical protein	Truncates by 31 aa	+	+	+	+	+	+	+	+
4769434	substitution	C→T	MAPK_4300	777	777	predicted acyl-CoA transferases/carnitine dehydratase	E→K								+
4772998	deletion	A	MAPK_4303	138	103	acyl dehydratase	Truncates by 35 aa					+			

SNPs identified within the seven human and bovine *Mycobacterium avium* subspecies *paratuberculosis* (MAP) isolate CLIJ623 compared to the K10 reference are shown. Position denotes the SNP coordinates within the reference genome K10. + represents the presence of the SNP within each isolate.

### Duplicated regions

**Figure 3:**
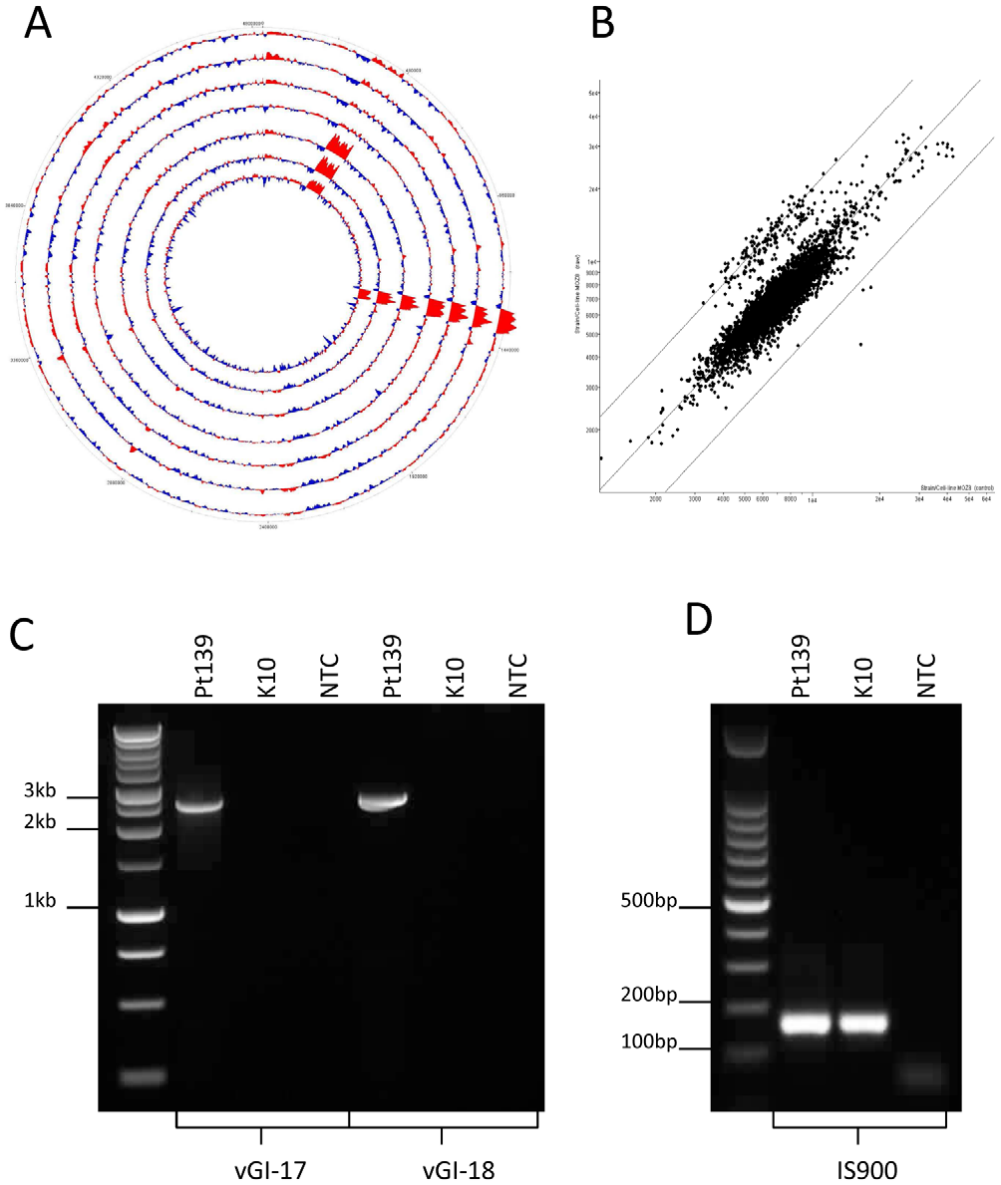
Schematics and PCR results for vGI-17 and vGI-18. A: Read depth plots for the seven human derived MAP isolates mapped onto the K10 reference genome. Regions with read depth exceeding the average are shown in *red*, while regions with below average depth are shown in *blue*. The inner track is Pt139, then Pt144, Pt145, Pt146, Pt154, Pt155 and the outer track is Pt164. B: MAPAC scatterplot comparing total genomic DNA from human MAP isolate Pt145 with MAP K10 control DNA. Diagonal lines represent −2, 0 and +2 fold differences in signal between test and control spots. vGI-17 and vGI-18 appear as clusters of significantly increased signal over MAP K10. C: PCR results from the human MAP isolate Pt139 and bovine isolate K10 for vGI-17 and vGI-18 duplications. D: IS*900* PCR as positive PCR control.

Two large genomic duplication regions were identified in some strains via their significantly higher sequencing coverage (Figure 3A). CGH also revealed an increase in signal intensity of approximately 2-fold when compared to K10 (Figure 3B). The first duplication, designated variable Genomic Island-17 (vGI-17), spanned 63 open reading frames including locus tags MAPK_1203 to MAPK_1265 (originally annotated as MAP2503 to MAP2565). Both sequence analysis and CGH showed this duplication to be present in all seven human derived isolates and the animal derived isolate CLIJ623. The second duplication, designated vGI-18, spanned 109 open reading frames including MAPK_0302 to MAPK_0410 (originally annotated as MAP3466 to MAP3358c). The vGI-18 duplication was only found to be present in three of the human derived isolates (Pt139, Pt144 and Pt145) revealed by sequence analysis and CGH. A full list of genes within vGI-17 and vGI-18 are included as Tables S3 and S4.

**Figure 4:**
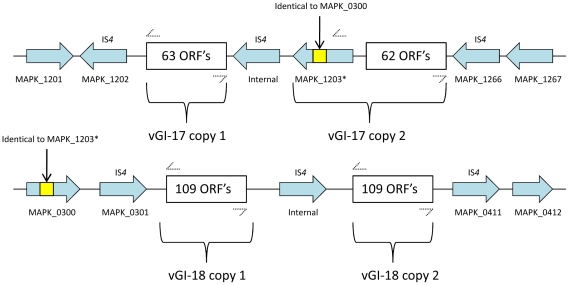
Genome organisation of vGI-17 and vGI-18. Each vGI consists of a tandem duplication separated by an internal IS*4* element. Approximate primer locations are indicated by *dashed* half arrows. *Yellow* boxes depict a 244 bp insertion into the duplicated copy of MAPK_1203 (designated MAPK_1203*). This sequence was 100% identical to a region of MAPK_0300 and only was found to be specific to ovine type I MAP strains. Refer to Table S3 and S4 for full lists of genes within vGI-17 and vGI-18.

Both vGI-17 and vGI-18 are flanked by copies of the IS*4* family transposase of which six copies are present in the K10 genome. Although this insertion sequence (IS) is annotated as an IS*4* family transposase, homology searches suggest it shows greater similarity to the IS*1182* family. However, until specific nomenclature is assigned to this IS, we will continue to use the IS*4* family annotation. A PCR based strategy was devised to confirm the position and orientation of vGI-17 and vGI-18 duplications. Outward oriented PCR primers were designed for both vGI-17 and vGI-18 located within the extreme 5′ and 3′ regions of the duplications and immediately internal of the adjacent IS*4* (Figure 4). Sequencing of PCR amplicons obtained with these primer pairs showed true tandem duplications of each region separated by an extra internal copy of the IS*4* family transposase (Figures 3C and 3D).

**Figure 5:**
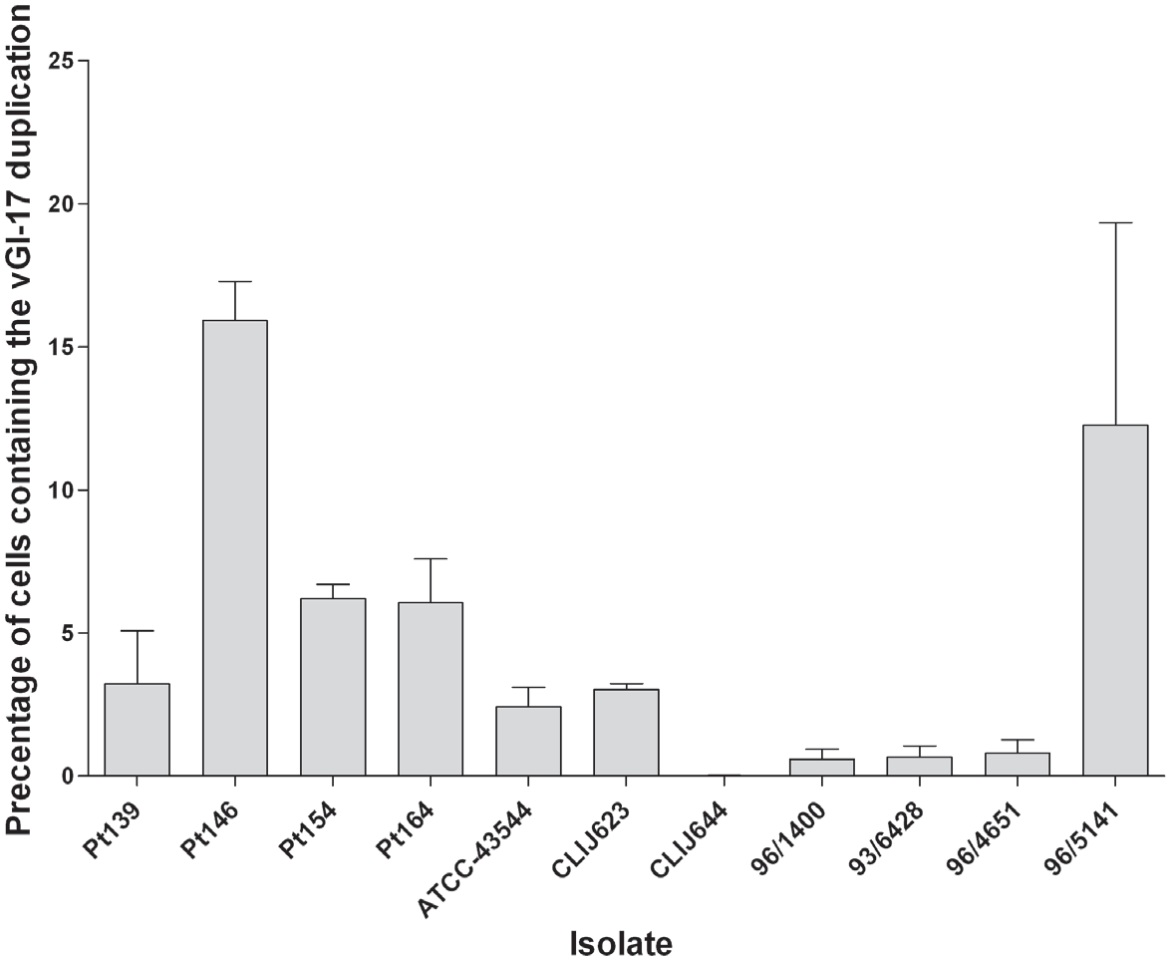
Proportion of cells containing vGI-17. Quantification of vGI-17 was determined for 11 isolates using a standard curve method. The copy number ratio of vGI-17 to the reference gene MAPK_3057 was used to determine the proportion of cells containing the vGI-17 duplication. Standard error bars were derived from triplicate PCR reactions.

To identify the frequency of tandem duplications in other MAP strains, duplication specific vGI-17 and vGI-18 PCRs were used to screen a panel of MAP isolates from a variety of hosts and geographical locations (Table 3). Both vGI-17 and vGI-18 tandem duplications were found in the majority of isolates examined, including those that which were not initially considered to contain duplications following genome sequencing and CGH. Indeed, vGI-17 and vGI-18 were identified as present in cultures of each of the three additional human isolates (ATCC-43544, ATCC43015 and SN8; refer to Table 1 for isolate descriptions) and many animal isolates including several geographical locations. The proportion of cells containing the vGI-17 duplication was quantified in a subset of isolates using real-time PCR. The absolute copy number of vGI-17 and the single copy endogenous reference gene MAPK_3057 was determined by the standard curve method against dilutions of recombinant plasmid and the ratio of vGI-17 to MAPK_3057 calculated as the proportion of total MAP cells in each culture (as a percentage) containing the vGI-17 duplication. This ranged between 0.01 to 15% (Figure 5) with human derived isolates containing a higher proportion of vGI-17 positive cells compared to bovine derived isolate with the exception of the bovine isolate 96/5141 and CLIJ623, which both had a comparable proportion of vGI-17 to human derived isolates. When quantified by real-time PCR the proportion of cells containing vGI-17 was lower than previously demonstrated by sequencing and CGH. We suggest this discrepancy may be a consequence of differences in culture age. Indeed isolates used for genome sequencing were grown for six to nine months whereas isolates for qPCR were cultured for approximately 20 months.

**Table 3:** vGI-17 and vGI-18 duplication PCR results.

Isolate	Type	Host	vGI-17	vGI-18
CLIJ623	II	bovine	+	+
CLIJ644	II	bovine	+	−
CLIJ361	I	ovine	−	−
Pt139	II	human	+	+
Pt144	II	human	+	+
Pt145	II	human	+	+
Pt146	II	human	+	+
Pt154	II	human	+	+
Pt155	II	human	+	+
Pt164	II	human	+	+
ATCC-19698	II	bovine	+	+
ATCC-43544 (Ben)	II	human	+	+
ATCC-43015 (Linda)	II	human	+	+
CLIJ684	II	bovine	+	+
CLIJ748	II	bovine	+	+
96/1400-1	II	bovine	+	+
93/6428	II	bovine	+	+
96/4651	II	bovine	+	+
96/5141	II	bovine	+	+
97/5541-1	I	ovine	+*	+
98/3368	I	ovine	+*	+
99/3759-2	I	ovine	+*	+
99/340	I	ovine	+*	+
K11	II	bovine	+	+
K18	II	bovine	−	−
K43	II	bovine	+	+
K57	II	bovine	+	−
K46	II	bovine	−	−
K47	II	bovine	+	+
K48	II	bovine	+	+
SN8	II	human	+	+
US23	II	bovine	+	−
456	II	caprine	−	−
464	II	caprine	−	−
213G	I pigmented	ovine	−	−
W43	II	-	+	−
S5	bison	caprine	+	−
G50	II	bovine	−	−
G128	II	bovine	+	−
DJ1	II	bovine	−	−
CAM87	III	caprine	−	−

PCR was performed on a panel of isolates derived from various host species and geographical locations.

*denotes a larger vGI-17 PCR product observed in some ovine MAP strains.

The PCR amplicon for vGI-17 was larger within the four ovine derived strains 97/5541-1, 98/3368, 99/3759-2 and 99/340. Sequence analysis of these products revealed that in this ovine strain a 244 bp region present in a gene immediately adjacent to vGI-18 (MAPK_0300 previously MAP3468) had been duplicated and replaced a 100 bp region within an ORF (MAPK_1265 previously MAP2502) immediately adjacent to vGI-17 (Figure 4). This result suggests both vGI-17 and vGI-18 are related and could indicate other genomic rearrangements associated with the IS*4* elements have occurred around these loci in the Type I MAP genome.

### Isolate sequences not present in K10

Sequence reads that did not align to the K10 reference genome were subsequently *de novo* assembled using Velvet 0.7.63 [35] and checked for contaminant sequences. These resultant contig sequences represent DNA present in the K10 genome. The ovine derived isolate CLIJ361 contained significantly more contigs compared to all other isolates sequenced (Table 4) totalling approximately 90 kb of unique sequence. The human and bovine derived isolates contained only a small number of unique contigs (Table 4).

**Table 4:** Summary of *de novo* assembly of unmapped reads for each isolate.

Isolate	Number of contigs^a^	Largest contig (bp)	Smallest contig (bp)
CLIJ623	1	113	-
CLIJ644	26	1128	85
CLIJ361	88	7694	100
Pt139	1	109	-
Pt144	4	175	123
Pt145	3	168	135
Pt146	2	143	107
Pt154	2	143	107
Pt155	11	328	122
Pt164	0	-	-

a Only contigs with sequence coverage above 10-fold were considered for further analysis.

**Figure 6:**
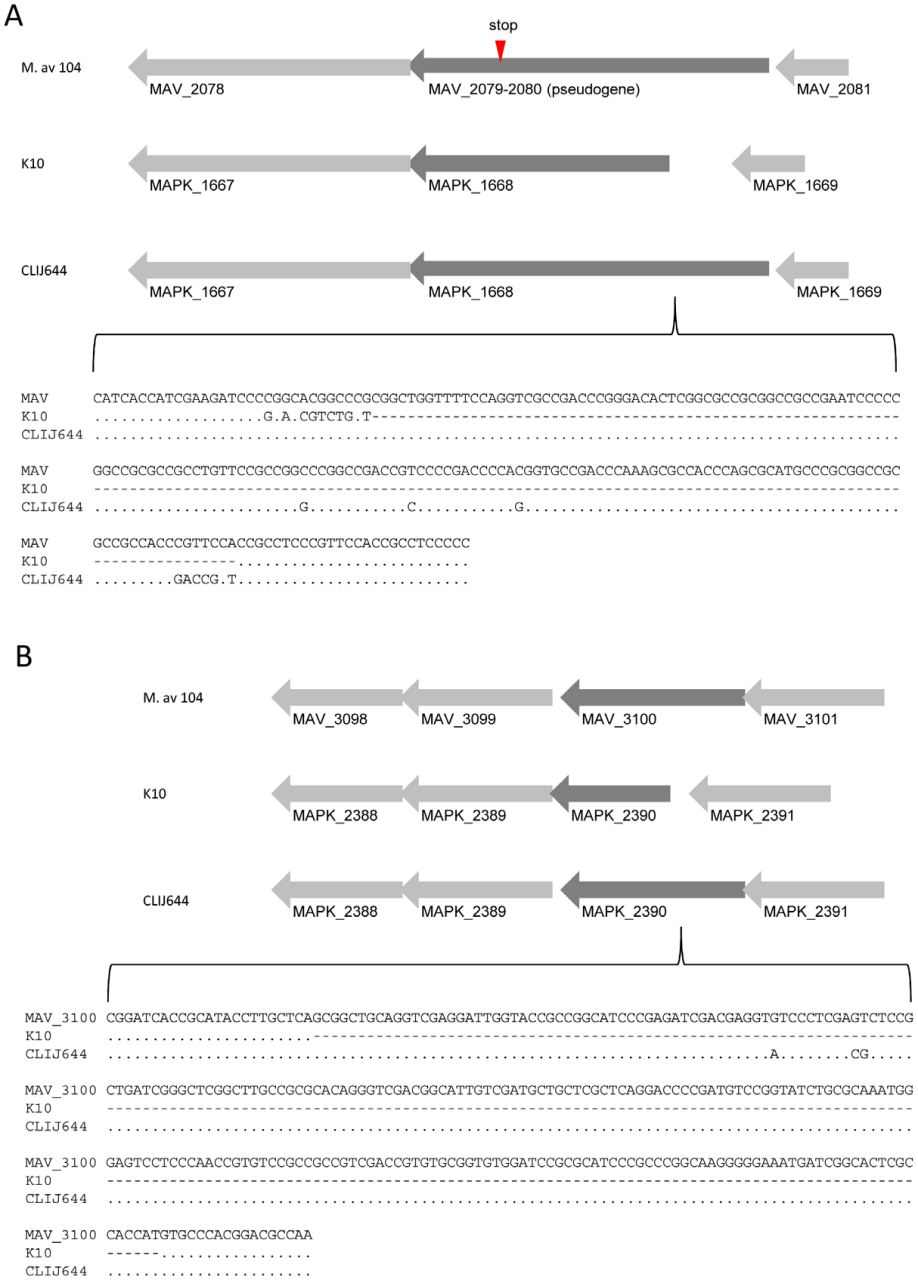
Schematic representation of genome insertions. (A) a 165 bp insertion up-stream of MAPK_1669 identified in all isolates compared to the K10 reference genome. The orthologous locus in *M. avium* subspecies *hominissuis* (M. av 104) is considered a pseudogene due to frame-shift induced a premature stop codon. (B) a 253 bp insertion up-stream of MAPK_2390 identified in bovine isolate CLIJ644 and ovine isolate CLIJ361.

All human and bovine (CLIJ623 and CLIJ644) derived isolates, but not the ovine derived isolate (CLIJ361), produced one common contig representing an additional 164 bp in the ABC-type multidrug transporter, MAPK_1668, compared to the K10 reference (Figure 6A). This extended the N-terminal region of the encoded protein by 173 aa relative to K10. This extended form resembles its ortholog in *M. avium* subspecies *hominissuis* (MAV_2079), and probably reflects a fixed deletion in the K10 strain rather than an identical insertion into both the Australian MAP strains and *M. avium* subspecies *hominissuis*.

Similarly, an additional 253 bp region located at the 5′ region of MAPK_2390 was identified and is predicted to encode a sulfotransferase (Figure 6B). This additional sequence extended the encoded protein sequence by 144 aa which more closely resembles its ortholog (MAV_3100) in *M. avium* subspecies *hominissuis*. This additional sequence was found only in bovine strain CLIJ644 and ovine strain CLIJ361.

All remaining *de novo* assembled contigs of bovine isolate CLIJ644 were found to be mapped to a single 5.3 kb region of the *M. avium* subspecies *hominissuis* genome. This region contained MAV_3107 (polyketide synthase) and MAV_3108 (erythronolide synthase, modules 3 and 4). Due to the repetitive nature of polyketide synthase genes, it remains unclear where exactly this 5.3 kb region is located within the CLIJ644 genome, however our data suggests it is likely to be between polyketide synthase Pks7 and Pks8. This additional sequence was unique to CLIJ644. A full description of contigs and their positions on the *M. avium* subspecies *hominissuis* genome is available as Table S5.

### K10 sequences not found in sequenced isolates

Read mapping identified regions of the K10 genome for which there was no corresponding sequence in the isolate from which the reads were derived. These unmapped regions represent differences in the coding capacity compared to K10. A total of 1574 unmapped regions were identified. At the sequence coverage used most of these unmapped regions were identified as regions with no or low sequence coverage. Conservative analysis identified four regions (two small and two large) that were present in K10 but not in one or more of the other strains. Read mapping of CLIJ361 revealed two large regions spanning 23 full and two partial open reading frames where no reads were mapped. The absence of this region compared to K10 has been reported previously in Type I MAP strains [17]. A 162 bp unmapped region within the hemolysin-like protein (MAPK_1064) was also observed within CLIJ361 as well as a 126 bp unmapped region within an acetyl-CoA acetyltransferase (MAPK_2970). Apart from small gaps in read depth, the human and bovine isolates did not contain any large unmapped regions compared to the K10 reference genome.

## Discussion

The aim of this study was to investigate the genetic relationship between multiple human and animal derived MAP strains at a genome-wide level. We hypothesised that genetic differences between strains may reveal phylogenetic relationships that provide a better understanding of the processes involved with MAP zoonotic transmission.

Our phylogenetic analysis based on SNPs showed a high genomic homogeneity within a panel of human isolates including four CD derived, a single UC derived and the two non-IBD control isolates. This is in agreement with the hypothesis that MAP, although capable of infecting humans, may not necessarily be able to trigger CD without cooperation from other factors influencing individual susceptibility. Significantly these human derived isolates were also closely related to a single dairy cattle isolate CLIJ623 [31] obtained within a similar geographical location (Victoria, Australia). This study only isolated a limited number of human strains and these were all MAP type II, but the relationship confirms previous studies demonstrating restricted allelic diversity within human MAP isolates [36] and a close genetic homogeneity to bovine isolates [21], [37]. This could also be indicative of a superior ability of bovine type II strains to persist within a human host. However, as Australians are much more likely to be exposed to MAP through bovine derived food products such as milk than ovine food products, this finding is more likely to be a reflection of the nature of zoonotic transmission and represents a common strain circulating amongst JD infected herds and humans in Victoria. The distinct genomic profile of MAP strains associated with humans determined in this work will allow future research to more fully investigate the geographical distribution and host preference of MAP human associated strains within Australian livestock species.

All MAP human associated isolates and the bovine isolate CLIJ623 were found to contain 24 non-synonymous SNPs. The majority of these result in single amino acid substitutions. However significant frame shifts occur in seven cases. The truncation by 149 aa of the acyl-CoA dehydrogenase FadE3_2 is particularly notable. The protein encoded by this gene has been shown to be significantly up-regulated *in vivo*-derived MAP from JD animals compared to laboratory cultured MAP [38] and also up-regulated in response to heat stress [39]. Furthermore the *M. avium* strain 905 also increased expression of proteins involved in fatty acid metabolism (FadE2) after phagocytosis by THP-1 cells [40]. The consequence of alterations in fatty acid metabolism for the survival of MAP in a human intracellular environment is as yet unknown, however FadE orthologs are known to be important in the catabolism of cholesterol by *Mycobacterium tuberculosis* during human cell entry [41] and so could be similarly involved in host specific carbon sourcing.

Gene duplication can increase genetic redundancy across potentially biologically important regions and thus may function to convey an enhanced ability of the organism to persist within a hostile environment. Duplication/deletion events involving IS elements have been reported previously in MAP [16]. Indeed, utilising CGH Castellanos et al. [16] detected 16 regions of consecutive genes (designated vGI-1 to vGI-16) with significantly altered signal ratios indicative of duplications/deletion. These polymorphisms were observed in both MAP Type I and MAP Type III strains and were often flanked by IS elements. Our study using both CGH and genome sequencing identifies two new large duplications (vGI-17 and vGI-18) spanning a total of 172 ORFs. Duplication specific PCR and amplicon sequencing confirmed that both vGI-17 and vGI-18 duplications are located directly in tandem in the genome, separated by an internal copy of the IS*4* family transposase and flanked by copies of the IS*4* family transposase. PCR screening of a panel of MAP strains from a worldwide selection of sources showed that vGI-17 and vGI-18 duplications were present at low proportion in the majority. Analysis of the vGI-17 duplication from ovine MAP isolates also suggested that an unusual form of this recombination/duplication event in these MAP types may have resulted in a fixed insertional event that has extended the reading frame of an ORF immediately adjacent to the duplication. The consequence of this is as yet unknown.

The organisation and duplication of IS*4* elements at the extremities of both vGI-17 and vGI-18 suggests that transposition/recombination may be the mechanism underlying the heterogeneity of duplication between MAP isolates. This study shows these duplications to be present within a proportion of cultures from most MAP isolates regardless of source location, host, and environment or strain type. Real-time PCR suggested that the proportion of cells containing the vGI-17 duplication in any single culture was significantly more prevalent in cultures of human derived strains compared to most other MAP isolates tested from a variety of sources. Variation in the abundance of vGI-17 in cultures appeared to decrease with age but could not be eliminated by single colony subculture. The propensity for clumping in MAP makes the generation of pure cultures problematic and could have influenced this however the variability between strains is indicative that these strains have capacity for inducible genomic plasticity. It remains unclear at this time if these are a result of mixed strain isolation from the primary source, differential induction or leaching during multiplication in an artificial culture environment or a predomination in primary cultures of human strains as a result of increased capacity for vGI duplication induced through adaptation to host environmental pressures and transmission cycles. Further work to determine the prevalence of vGI-17 and vGI-18 duplications within *in vivo* derived MAP is thus called for.

Duplications have been observed in other *Mycobacterium* species. Interestingly, a proportion of the genes within vGI-18 are also duplicated within the genome of *Mycobacterium bovis* BCG [42]. This finding suggests a conserved regulation of specific duplications may exist within the Mycobacterium genus. In *M. bovis* BCG the DU2 tandem duplication occurs as one of four different forms with an overlap of three intact genes between variants. One of these overlapping genes is *glpD2* (glycerol-3-phosphate dehydrogenase) located within the centre of vGI-18 (MAPK_0345). Brosch et al. [42] suggested that duplication of the *glpD2* locus may enhance growth on glycerol based media. A glycerol rich (Herrold's) culture medium was used to grow our MAP strains so it is possible that duplication of *glpD2* and its surrounding loci may have been promoted within this environment. A most intriguing aspect of vGI-17 is the duplication of the transcriptional regulator sigma factor E (*sigE*) which controls a regulon of genes essential for intracellular survival and virulence. In *M. tuberculosis* the *sigE* regulon promotes intracellular survival through mediation of the host inflammatory response [43]. In addition, other members of the sigma transcription factor family, namely *sigF* and *sigJ*, were also found to be duplicated within vGI-18. Within *Mycobacterium bovis*, the expression of *sigF* has been found to be up-regulated in response to a variety of stresses, including antibiotic stress, nutrient depletion and oxidative stress [44]. Similarly, the expression of *sigJ* in *M. tuberculosis* is also induced by oxidative stress [45]. It is tempting to hypothesise that MAP strains that contain multiple copies of the *sigE*, *sigJ* and *sigF* transcriptional regulators may exhibit superior ability to mediate the host inflammatory and stress responses, which, in turn, may impart a superior ability to persist with the intracellular environment. However, considering that the anti-sigma E factor (*htrA*) is located immediately downstream of *sigE* within vGI-17, as well as the anti-sigma F factor (*rsbW*) within vGI-18, it remains unclear whether an additional copy of the sigma factors in the presence of an additional copy of its anti-sigma regulator would have any transcriptional effect compared to a strain with single copies of both. Further work is in progress to investigate these relationships and the influences triggering duplication events.

Other duplicated genes with known biological relevance include the two-component signal transduction system *mtrA* and *mtrB* (MAPK_0408-0409), known to be an essential for intracellular survival and infectivity of *M. tuberculosis* [46], [47] and a number of tricarboxylic acid cycle enzymes within vGI-17 and vGI-18 including succinate dehydrogenase, isocitrate dehydrogenase, malate dehydrogenase, malate oxidoreductase and fumarate reductase. The duplication of these loci may act to enhance survival and metabolic capacity of MAP within intracellular environments.

In conclusion this study provides genomic evidence of MAP zoonotic transmission from domestic animals to humans. Mutations in over 71 distinct loci [48], [49] are currently linked to CD, many having functions related to the processing of intracellular pathogens. This link suggests that intracellular pathogens such as MAP may play a role in the disease progression of CD patients who have pathogen specific susceptibility genes. The presence in humans of an inductive MAP phenotype discovered in this study may offer significant insights for future work. Research within our laboratories is now concentrating on determining the functional relevance and regulation of large region duplications in conjunction with a molecular epidemiological survey of MAP strains to determine their frequency of occurrence in animal hosts.

## Supporting Information

Table S1 Primers used in this study.
(DOC)

Table S2 The position and nature of polymorphism is described for all core SNPs identified within this study. SNPs were detected when compared to the reference strain K10. The frequency of nucleotides at the SNP region is also reported.
(XLS)

Table S3 List of 63 genes duplicated within vGI-17. Clusters of Orthologous Groups (COGs) have also been annotated for each genes corresponding protein sequence.
(DOC)

Table S4 List of 109 genes duplicated within vGI-18. Clusters of Orthologous Groups (COGs) have also been annotated for each genes corresponding protein sequence. *Grey* cells denote loci which are also duplicated with *Mycobacterium bovis* BCG.
(DOC)

Table S5 A full description of contigs and their positions on the *M. avium* subspecies *hominissuis* genome which are unique to the bovine isolate CLIJ644 compared to the K10 reference.
(XLS)
