# Supplementary material for: Exploring the Zoonotic Potential of Mycobacterium avium Subspecies paratuberculosis through Comparative Genomics
Source: PLoS One. 2011 Jul 22;6(7):e22171. doi: 10.1371/journal.pone.0022171 (PMC3142125; doi:10.1371/journal.pone.0022171)
Supplement: Table S1 — Primers used in this study. (DOC) [file pone.0022171.s001.doc]

| **Primer name** | **Sequence (5’-3’)** | **Purpose** |
| --- | --- | --- |
| JF11 | ACGGTTACGGAGGTGGTTGTGG | IS*900* |
| JF12 | CTCCATCGGCCAACGTCGTCAC | IS*900* |
| GSP1-5’-vGI-17 | CATCGGGGAACTACGCGAAATACTGTC | vGI-17 duplication |
| GSP1-3’-vGI-17 | CTTGCGCAGGTTGGATACGTAGGAGTG | vGI-17 duplication |
| GSP1-5’- vGI-18 | CTACCTGCTGGCCAAGATCGTGGTGTT | vGI-18 duplication |
| GSP1-3’- vGI-18 | ACATCGTGGTGACTCGCACCGACTCCA | vGI-18 duplication |
| MAPK_3057F | GACTACGACGCGCTCAAA | qPCR control |
| MAPK_3057R | GTCCGAGGCGAAGAACAG | qPCR control |

**Supplementary Table 1; Primers used in this study.**
