# Supplementary material for: Exploring the Zoonotic Potential of Mycobacterium avium Subspecies paratuberculosis through Comparative Genomics
Source: PLoS One. 2011 Jul 22;6(7):e22171. doi: 10.1371/journal.pone.0022171 (PMC3142125; doi:10.1371/journal.pone.0022171)
Supplement: Table S3 — List of 63 genes duplicated within vGI-17. Clusters of Orthologous Groups (COGs) have also been annotated for each genes corresponding protein sequence. (DOC) [file pone.0022171.s003.doc]

Table S3. List of 63 genes duplicated within vGI-17. Clusters of Orthologous Groups (COGs) have also been annotated for each genes corresponding protein sequence.

| **Locus_tag** | **Product** | **COG assignment** |
| --- | --- | --- |
| MAPK_1203 | glycogen synthase | Cell envelope biogenesis, outer membrane |
| MAPK_1204 | glucose-1-phosphate adenylyltransferase | Carbohydrate transport and metabolism |
| MAPK_1205 | Predicted acyl esterases | General function prediction only |
| MAPK_1206 | Putative protein-S-isoprenylcysteine methyltransferase | Function unknown |
| MAPK_1207 | Putative exporter of polyketide antibiotics | Cell envelope biogenesis, outer membrane |
| MAPK_1208 | ABC-type multidrug transport system, ATPase component | Defense mechanisms |
| MAPK_1209 | Transcriptional regulator | Transcription |
| MAPK_1210 | Predicted O-methyltransferase | General function prediction only |
| MAPK_1211 | alternative RNA polymerase sigma factor SigE | Transcription |
| MAPK_1212 | conserved hypothetical protein | No COG |
| MAPK_1213 | serine protease htrA | Posttranslational modification, protein turnover, chaperones |
| MAPK_1214 | Sec-independent protein secretion pathway components | Intracellular trafficking and secretion |
| MAPK_1215 | MRP-family ATP-binding protein | Cell division and chromosome partitioning |
| MAPK_1216 | Membrane-bound lytic murein transglycosylase B | Cell envelope biogenesis, outer membrane |
| MAPK_1217 | Predicted membrane protein | Function unknown |
| MAPK_1218 | Mg/Co/Ni transporter MgtE (contains CBS domain) | Inorganic ion transport and metabolism |
| MAPK_1219 | conserved hypothetical protein | No COG |
| MAPK_1220 | sugar-binding lipoprotein LpqY | Carbohydrate transport and metabolism |
| MAPK_1221 | ABC transporter, permease protein SugA | Carbohydrate transport and metabolism |
| MAPK_1222 | ABC transporter, permease protein SugB | Carbohydrate transport and metabolism |
| MAPK_1223 | ABC transporter, ATP-binding protein SugC | Carbohydrate transport and metabolism |
| MAPK_1224 | conserved hypothetical protein | No COG |
| MAPK_1225 | conserved hypothetical protein | No COG |
| MAPK_1226 | magnesium and cobalt transport protein CorA | Inorganic ion transport and metabolism |
| MAPK_1227 | malate dehydrogenase | Energy production and conversion |
| MAPK_1228 | malate oxidoreductase | Energy production and conversion |
| MAPK_1229 | lipoprotein LpqZ | Cell envelope biogenesis, outer membrane |
| MAPK_1230 | Short-chain alcohol dehydrogenase of unknown specificity | General function prediction only |
| MAPK_1231 | Short-chain alcohol dehydrogenase of unknown specificity | General function prediction only |
| MAPK_1232 | 2-oxoglutarate dehydrogenase sucA | Energy production and conversion |
| MAPK_1233 | conserved hypothetical protein | No COG |
| MAPK_1234 | Arabinose efflux permease | Carbohydrate transport and metabolism |
| MAPK_1235 | Predicted nuclease (RecB family) | DNA replication, recombination, and repair |
| MAPK_1236 | Cytochrome P450 | Secondary metabolites biosynthesis, transport, and catabolism |
| MAPK_1237 | conserved hypothetical protein | No COG |
| MAPK_1238 | short chain dehydrogenase | Secondary metabolites biosynthesis, transport, and catabolism |
| MAPK_1239 | conserved hypothetical protein | Function unknown |
| MAPK_1240 | carveol dehydrogenase | Secondary metabolites biosynthesis, transport, and catabolism |
| MAPK_1241 | Transcriptional regulator | Transcription |
| MAPK_1242 | 3-oxoacyl-(acyl-carrier-protein) reductase | Secondary metabolites biosynthesis, transport, and catabolism |
| MAPK_1243 | Cytochrome P450 | Secondary metabolites biosynthesis, transport, and catabolism |
| MAPK_1244 | Coenzyme F420-dependent N5,N10-methylene tetrahydromethanopterin reductase | Energy production and conversion |
| MAPK_1245 | Uncharacterized conserved protein related to dihydrodipicolinate reductase | Function unknown |
| MAPK_1246 | lipoprotein LprE | No COG |
| MAPK_1247 | cold-shock DEAD-Box protein, DeaD | DNA replication, recombination, and repair |
| MAPK_1248 | Predicted acyltransferases | Lipid metabolism |
| MAPK_1249 | Transcriptional regulator | Transcription |
| MAPK_1250 | Cytochrome P450 | Secondary metabolites biosynthesis, transport, and catabolism |
| MAPK_1251 | glycolate oxidase, subunit GlcD | Energy production and conversion |
| MAPK_1252 | conserved hypothetical protein | No COG |
| MAPK_1253 | uracil-DNA glycosylase | DNA replication, recombination, and repair |
| MAPK_1254 | 2-polyprenyl-6-methoxyphenol hydroxylase and related FAD-dependent oxidoreductases | Energy production and conversion |
| MAPK_1255 | Coenzyme F420-dependent N5,N10-methylene tetrahydromethanopterin reductase | Energy production and conversion |
| MAPK_1256 | diguanylate cyclase/phosphodiesterase | Signal transduction mechanisms |
| MAPK_1257 | cyclic diguanylate phosphodiesterase domain-containing protein | No COG |
| MAPK_1258 | HIT family protein | Nucleotide transport and metabolism |
| MAPK_1259 | amidase AmiB2 | Translation, ribosomal structure and biogenesis |
| MAPK_1260 | G:T/U mismatch-specific DNA glycosylase | DNA replication, recombination, and repair |
| MAPK_1261 | Adenylate cyclase, family 3 (some proteins contain HAMP domain) | Signal transduction mechanisms |
| MAPK_1262 | conserved hypothetical protein | No COG |
| MAPK_1263 | conserved hypothetical protein | No COG |
| MAPK_1264 | serine-threonine protein kinase | Signal transduction mechanisms |
| MAPK_1265 | transcriptional regulatory protein EmbR2 | Signal transduction mechanisms |
