# Supplementary material for: Exploring the Zoonotic Potential of Mycobacterium avium Subspecies paratuberculosis through Comparative Genomics
Source: PLoS One. 2011 Jul 22;6(7):e22171. doi: 10.1371/journal.pone.0022171 (PMC3142125; doi:10.1371/journal.pone.0022171)
Supplement: Table S4 — List of 109 genes duplicated within vGI-18. Clusters of Orthologous Groups (COGs) have also been annotated for each genes corresponding protein sequence. Grey cells denote loci which are also duplicated with Mycobacterium bovis BCG. (DOC) [file pone.0022171.s004.doc]

Table S4. List of 109 genes duplicated within vGI-18. Clusters of Orthologous Groups (COGs) have also been annotated for each genes corresponding protein sequence. *Grey* cells denote loci which are also duplicated with *Mycobacterium bovis* BCG.

| **Locus_tag** | **Product** | **COG assignment** |
| --- | --- | --- |
| MAPK_0302 | ABC-type multidrug transport system, ATPase component | Defense mechanisms |
| MAPK_0303 | ABC-type multidrug transport system, ATPase component | Defense mechanisms |
| MAPK_0304 | oxidoreductase, FMN-binding | Energy production and conversion |
| MAPK_0305 | bifunctional protein: FolD | Coenzyme metabolism |
| MAPK_0306 | conserved hypothetical membrane protein | No COG |
| MAPK_0307 | conserved hypothetical protein | No COG |
| MAPK_0308 | conserved hypothetical protein | No COG |
| MAPK_0309 | methyltransferase | Secondary metabolites biosynthesis, transport, and catabolism |
| MAPK_0310 | homoserine O-acetyltransferase metA | Amino acid transport and metabolism |
| MAPK_0311 | O-acetylhomoserine sulfhydrylase metC | Amino acid transport and metabolism |
| MAPK_0312 | monomeric isocitrate dehydrogenase icd2 | Energy production and conversion |
| MAPK_0313 | isocitrate dehydrogenase icd1 | Energy production and conversion |
| MAPK_0314 | conserved hypothetical protein | No COG |
| MAPK_0315 | tryptophanyl-tRNA synthetase trpS | Translation, ribosomal structure and biogenesis |
| MAPK_0316 | ribonuclease, BN-like | Replication, recombination, and repair |
| MAPK_0317 | multidrug resistance protein, SMR family protein | No COG |
| MAPK_0318 | N-acetylglucosamine-6-phosphate deacetylase nagA | Carbohydrate transport and metabolism |
| MAPK_0319 | sugar-transport integral membrane protein SugI | Carbohydrate transport and metabolism |
| MAPK_0320 | penicillin-binding protein dacB | Cell envelope biogenesis, outer membrane |
| MAPK_0321 | adenosylmethionine-8-amino-7-oxononanoate aminotransferase | Coenzyme metabolism |
| MAPK_0322 | alternative RNA polymerase sigma factor SigJ | Transcription |
| MAPK_0323 | conserved hypothetical protein | No COG |
| MAPK_0324 | succinate dehydrogenase (iron-sulphur protein subunit) SdhB | Energy production and conversion |
| MAPK_0325 | succinate dehydrogenase (flavoprotein subunit) SdhA | Energy production and conversion |
| MAPK_0326 | succinate dehydrogenase (hydrophobic membrane anchor subunit) SdhD | Energy production and conversion |
| MAPK_0327 | succinate dehydrogenase (cytochrome B-556 subunit) SdhC | Energy production and conversion |
| MAPK_0328 | cytidine deaminase Cdd | Nucleotide transport and metabolism |
| MAPK_0329 | thymidine phosphorylase DeoA | Nucleotide transport and metabolism |
| MAPK_0330 | adenosine deaminase Add | Nucleotide transport and metabolism |
| MAPK_0331 | transcription regulator | Transcription |
| MAPK_0332 | conserved hypothetical protein | No COG |
| MAPK_0333 | conserved hypothetical protein | No COG |
| MAPK_0334 | conserved hypothetical protein | No COG |
| MAPK_0335 | conserved hypothetical protein | No COG |
| MAPK_0336 | acid phosphatase | No COG |
| MAPK_0337 | uracil phosphoribosyltransferase Upp | Nucleotide transport and metabolism |
| MAPK_0338 | phosphomannomutase PmmB | Carbohydrate transport and metabolism |
| MAPK_0339 | purine nucleoside phosphorylase DeoD | Nucleotide transport and metabolism |
| MAPK_0340 | cutinase | No COG |
| MAPK_0341 | amidohydrolase amiB | General function prediction only |
| MAPK_0342 | N-acyl-L-amino acid amidohydrolase amiA1 | General function prediction only |
| MAPK_0343 | conserved hypothetical protein | No COG |
| MAPK_0344 | dihydrolipoamide dehydrogenase LpdA | Energy production and conversion |
| MAPK_0345 | glycerol-3-phosphate dehydrogenase GlpD2 | Energy production and conversion |
| MAPK_0346 | RNA pseudouridylate synthase family protein | Translation, ribosomal structure and biogenesis |
| MAPK_0347 | Superfamily II RNA helicase | No COG |
| MAPK_0348 | PPE-repeat proteins | Cell motility and secretion |
| MAPK_0349 | PPE-repeat proteins | Cell motility and secretion |
| MAPK_0350 | conserved hypothetical secreted protein | No COG |
| MAPK_0351 | esterase lipoprotein LpqC | Secondary metabolites biosynthesis, transport, and catabolism |
| MAPK_0352 | endonuclease VIII Nei | DNA replication, recombination, and repair |
| MAPK_0353 | ATP-dependent helicase lhr | General function prediction only |
| MAPK_0354 | transcriptional regulatory protein (probably TetR-family) | Transcription |
| MAPK_0355 | aldehyde dehydrogenase (NAD) family protein | Energy production and conversion |
| MAPK_0356 | conserved hypothetical protein | No COG |
| MAPK_0357 | transcriptional regulatory protein (probably AsnC-family) | Transcription |
| MAPK_0358 | L-lysine-epsilon aminotransferase lat | Amino acid transport and metabolism |
| MAPK_0359 | conserved hypothetical protein | No COG |
| MAPK_0360 | conserved hypothetical protein | No COG |
| MAPK_0361 | anti-sigma factor RsbW | Signal transduction mechanisms |
| MAPK_0362 | alternate RNA polymerase sigma factor SigF | Transcription |
| MAPK_0363 | STAS domain-containing protein (anti-sigma-factor antagonist) | Signal transduction mechanisms |
| MAPK_0364 | bifunctional protein acetyl-/propionyl-coenzyme a carboxylase (alpha chain) accA3 | Lipid metabolism |
| MAPK_0365 | Fe-S metabolism associated domain subfamily | Posttranslational modification, protein turnover, chaperones |
| MAPK_0366 | thiosulfate sulfurtransferase sseA | Inorganic ion transport and metabolism |
| MAPK_0367 | maf protein | Cell division and chromosome partitioning |
| MAPK_0368 | conserved hypothetical protein | No COG |
| MAPK_0369 | propionyl-CoA carboxylase accD5 | Lipid metabolism |
| MAPK_0370 | Succinate dehydrogenase/fumarate reductase, flavoprotein subunit | General function prediction only |
| MAPK_0371 | bifunctional protein birA | Coenzyme metabolism |
| MAPK_0372 | conserved hypothetical protein | No COG |
| MAPK_0373 | conserved hypothetical protein | No COG |
| MAPK_0374 | phosphoribosylaminoimidazole carboxylase ATPase subunit purK | Nucleotide transport and metabolism |
| MAPK_0375 | phosphoribosylaminoimidazole carboxylase catalytic subunit purE | Nucleotide transport and metabolism |
| MAPK_0376 | acyl-CoA dehydrogenase | Lipid metabolism |
| MAPK_0377 | Serine phosphatase RsbU, regulator of sigma subunit | Signal transduction mechanisms |
| MAPK_0378 | two component system membrane associated sensor kinase | Signal transduction mechanisms |
| MAPK_0379 | Response regulator containing a CheY-like receiver domain | Signal transduction mechanisms |
| MAPK_0380 | phosphate ABC transporter, phosphate-binding protein pstS | Inorganic ion transport and metabolism |
| MAPK_0381 | serine/threonine-protein kinase pknD | Signal transduction mechanisms |
| MAPK_0382 | acyl-CoA transferase | Energy production and conversion |
| MAPK_0383 | O-Methyltransferase involved in polyketide biosynthesis | Secondary metabolites biosynthesis, transport, and catabolism |
| MAPK_0384 | metal cation-transporting p-type ATPase C ctpC | Inorganic ion transport and metabolism |
| MAPK_0385 | conserved hypothetical protein | No COG |
| MAPK_0386 | conserved hypothetical protein | No COG |
| MAPK_0387 | cell envelope-related function transcriptional attenuator | Transcription |
| MAPK_0388 | dTDP-4-dehydrorhamnose reductase rmlD | Cell envelope biogenesis, outer membrane |
| MAPK_0389 | dTDP-rha:A-D-GlcNAc-diphosphoryl polyprenol A-3-L-rhamnosyl transferase, wbbL | Cell envelope biogenesis, outer membrane |
| MAPK_0390 | d-alpha-D-mannose-1-phosphate guanylyltransferase manB | Cell envelope biogenesis, outer membrane |
| MAPK_0391 | conserved hypothetical protein | No COG |
| MAPK_0392 | DNA methylase | DNA replication, recombination, and repair |
| MAPK_0393 | F420-0--gamma-glutamyl ligase | Coenzyme transport and metabolism |
| MAPK_0394 | LPPG:Fo 2-phospho-L-lactate transferase | Function unknown |
| MAPK_0395 | hypothetical protein | No COG |
| MAPK_0396 | transcription factor whiB | Transcription |
| MAPK_0397 | conserved hypothetical protein | No COG |
| MAPK_0398 | conserved hypothetical protein | No COG |
| MAPK_0399 | phosphomannomutase pmmA | Carbohydrate transport and metabolism |
| MAPK_0400 | conserved hypothetical protein | No COG |
| MAPK_0401 | mannose-6-phosphate isomerase manA | Carbohydrate transport and metabolism |
| MAPK_0402 | conserved hypothetical protein | No COG |
| MAPK_0403 | cationic amino acid transporter | Amino acid transport and metabolism |
| MAPK_0404 | alkane-1-monooxygenase | Energy production and conversion |
| MAPK_0405 | conserved hypothetical protein | No COG |
| MAPK_0406 | adenosylhomocysteinase sahH | Coenzyme metabolism |
| MAPK_0407 | thymidylate kinase tmk | Nucleotide transport and metabolism |
| MAPK_0408 | two component sensory transduction transcriptional regulatory protein mtrA | Signal transduction mechanisms |
| MAPK_0409 | two component sensory transduction histidine kinase mtrB | Signal transduction mechanisms |
| MAPK_0410 | conserved lipoprotein lpqB | No COG |
